# Exercise motivation and quality of life among the elderly during the COVID-19 pandemic

**DOI:** 10.25122/jml-2021-0185

**Published:** 2022-04

**Authors:** Muria Herlina

**Affiliations:** 1. Department of Social Welfare, School of Social and Political Sciences, Universitas Bengkulu, Sumatera, Indonesia

**Keywords:** elderly, exercise, health, motivation, quality of life

## Abstract

This study aimed to investigate the motivation of participating in exercise and the impact on the quality of life among the elderly during the Covid-19 pandemic. This study used a mixed-method (quantitative and qualitative) and included 109 respondents (72 women and 37 men) to answer the questionnaires. In addition, the study included 15 participants for the qualitative phase, consisting of 8 women and 5 men from the elderly group, 1 village official, and 1 youth leader. The results showed a significant correlation between the motivation of following exercise and the quality of life among elderly.

## Introduction

Everyone expects to live a quiet life and enjoy a peaceful old age. Growing old is unavoidable, and it is a natural process. The elderly are people who have reached the age of 60 years and over. In Constitutions no. 13 of 1998 in Chapter 1 Article 1, Paragraphs 2 and 3, concerning the welfare of elderly people, there are two categories of elderly, namely potential elderly and non-potential elderly [1]. 

The United Nations reported that Indonesia is one of the countries with the highest explosion in older people globally, with around 414% over 35 years from 1990 to 2025. In 2025, it is estimated that Indonesia will have the fifth largest population of elderly people after China, India, the United States, and Japan [2]. Between 2004 and 2015, Indonesia had an increase in life expectancy from 68.6 years to 70.8 years, and projections for 2030–2035 reach 72.2 years. More than two-thirds of the elderly live in rural areas, as generally happens in developing countries. Based on the population census in 2010, the number of elderly people in Indonesia reached 18.1 million people, or 7.6% of the total population. In 2014, the number of elderly people increased to 18.781 million, and in 2025, the number is estimated to be 36 million. According to the World Health Organization Quality of Life (WHOQOL), the quality of life can be assessed from the fulfillment of physical health, psychological health, social relationship, and environmental conditions [3].

One of the efforts of the Indonesian government is to maintain and improve elderly's health through exercise. The increase in the incidence of disease among elderly people can affect their quality of life [4]. When entering old age, the function of the organs in the body becomes reduced, and it can be said that they are unable to function properly. Thus, elderly people need a lot of help in carrying out their activities [5]. Various diseases that appear with old age make older people need extra attention. Doing an exercise will help their body become fit and fresh because it trains bones to stay strong, encourages the heart to work optimally, and helps to eliminate free radical roaming in the body. In other words, they will have good physical fitness. If the body has good blood and heart circulation, the whole body can carry out its function for a long time [6]. The quality of elderly life can be improved by adopting a healthy lifestyle, such as physical activities. Exercise has benefits, including strengthening muscles and joints, improving blood circulation, reducing the risk of the disease such as heart disease, stroke, type 2 diabetes, maintaining brain health and function while reducing the risk of brain disorders such as dementia, reducing stress and risk of mental disorders [7]. Elderly exercising regularly can also remain productive and live more independently.

The regulation of the Minister of Health of the Republic of Indonesia Number 21 of 2020, Article 2 of the strategic plan of the Ministry of Health 2020–2024, must be used as a reference for all work units within the Ministry of Health [8]. The purpose of Sustainable Development Goals (SDG) is an international commitment to improve the quality of life from one generation to the next. The RPJMN (Medium-Term National Development Plan) 2020–2024 policy related to the purpose of Sustainable Development Goals (SDG) discusses 17 purposes, including three points which state that a healthy and prosperous life is (1) controlling population growth and strengthening population governance, (2) strengthening the implementation of social protection and (3) increasing health services towards universal health coverage, especially the strengthening of basic health services (Primary Health Care) [9]. This description includes the declaration of elderly health. 

The reality of the elderly's life in Indonesia, especially in Bengkulu Province, is that they experience a lack of motivation, so they tend to stay at home and not do physical activity. Most elderly people do not perform physical activity such as regular exercise [10]. The inhibiting factors include the lack of encouragement from the family and the unavailability of facilities such as exercise coaches for elderly people. Meanwhile, Wang *et al.* argued that someone with high socio-economic status has a stronger motivation to perform health-related behaviors and maintain a healthy lifestyle to reduce health issues, thereby increasing health provisions in old age [11]. A study in Florence, Italy, stated that socio-economic status and health literacy (HL) positively relate [12]. This finding confirmed the need to explore the role of potential mediation from HL in the relationship between socioeconomic factors and health. 

## Material and Methods

This study was conducted for three months in August, September, and November 2020, in three areas: Pembatu Health Center, Bitunan Village, Batik Nau village, and Sukarga Village in North Bengkulu, Indonesia. This study used a mixed-method qualitative and quantitative approach because of its several advantages. Quantitatively, this paper is a correlational study that only shows the relationship between variables. However, qualitatively, all answers about the relationship can be found and mutually support the data [13, 14]. To determine the correlation between variables, the data were quantitatively collected using a questionnaire distributed to 109 elderly consisting of 72 elderly women and 37 elderly men, who were obtained using the Slovin formula: [14]. Also, quantitative data were analyzed using a t-test and Chi-Square test using SPSS 21 [15]. 

The elderly's intrinsic and extrinsic motivation in participating in the exercise was classified into two categories: low category (<50%) and high category (>50%). Quality of life was measured using four indicators, *i.e.*, physical health, psychological health, social health, and environmental health. If more than two indicators were met, the quality of life was categorized as high; if there were less than two indicators, the quality of life was low. The qualitative approach was used to understand the social phenomenon and emphasize a complete picture to gain a deeper understanding [16, 17]. 

The data were obtained through in-depth interviews, focus group discussions (FGD), and observations [17]. FGD can be interpreted as a discussion that is carried out systematically and focused on a particular issue or problem [18]. The interview was conducted and recorded using a semi-structured interview guide (guided interview). It aimed to reveal information about experiences in a person's life to find in-depth experiences according to the research topic. The informants were obtained through sampling using the criteria: (1) elderly living for at least five years in three predetermined villages, (2) attending elderly exercise at an elderly Posyandu (a community-based activity for health services) for at least the last one year, (3) understanding and able to discuss in Indonesian or local languages (Rejang language), and (4) willing to explain information according to the research topic. The FGD was conducted two times, with a group of eight people each, since they were limited in number, and it was challenging to gather them due to their old age. The FGD aimed to explore their opinions about the norms of the elderly group in a short time. 

The observations were carried out in an unstructured manner to note the behavior of the elderly as a whole, such as their living conditions, residences, participation in the elderly exercise, and Posyandu condition. The research target was expected to have 15 informants, 8 women and 5 men from the elderly group, 1 official from the village, and 1 youth leader representative. They were all involved until the results of the in-depth interviews were saturated. For this reason, the in-depth interviews were stopped [17].

Reliability in qualitative research is different from quantitative research due to their different paradigms in seeing reality. Qualitative research is multiple and dynamic, and its reliability test is internal validity. In addition, since the beginning, qualitative researchers have analyzed data by mingling with research subjects and using the narrative form [13]. A qualitative approach uses a matrix table to draw conclusions [14].

According to data taken from Bengkulu Province Central Statistics Agency, as cited by Syahputra, the number of elderly people in Bengkulu is as many as 123,871 people, including ten regencies and one city [19]. Most elderly are in North Bengkulu Regency, 19,644 people, and the fewest is in Kaur Regency, 15,367 people. This is one of the reasons why North Bengkulu was the research location.

## Results

### Univariate analysis

The result from Table 1 shows that the intrinsic motivation of the elderly was relatively high. Of 109 elderly people, 84 people were encouraged to participate, while 25 were not interested in exercise. The result was supported by a statement from a village official, AN, 41 years old, SLTA. 

**Table 1.** Intrinsic motivation to take part in elderly exercise.

**Motivation 1**				
**Valid**	**Frequency**	**Percent**	**Valid percent**	**Cumulative percent**
**Low**	25	22.9	22.9	22.9
**High**	84	77.1	77.1	100.0
**Total**	109	100.0	100.0	

*“There are many elderly people in the Batik Nau sub-district; North Bengkulu perform exercises based on their own will; some get encouragement from outside, such as support from their children, neighbors, or grandchildren who are willing to take them to an exercise studio at Pustu”.*

The study conducted by Alvita and Sholihul in Jepara Public Health Center, Central Java, stated that exercise improves elderly skills to prevent body balance disorder [20]. Elderly exercise can help to reduce blood pressure for those who suffer from hypertension [21]. According to Guo *et al.*, middle-aged people and elderly people in China who have diabetes do some physical activities such as exercise. It is essential to improve their health to avoid economic burden in their future life [22]. 

Table 2 shows that from 109, about 71 elderly people were encouraged to exercise at home because of the Covid-19 pandemic. Meanwhile, as many as 38 people were not interested in doing exercise or had low motivation. Therefore, it shows that the extrinsic motivation of elderly people to take part in exercise was relatively high. Extrinsic motivation itself is an urge to do something which comes from outside. ST, 71 years old, and RD, 63 years old, mentioned:

**Table 2.** Extrinsic motivation to take part in elderly exercise.

**Motivation 2**					
		**Frequency**	**Percent**	**Valid percent**	**Cumulative percent**
**Valid**	**Low**	38	34.9	34.9	34.9
	**High**	71	65.1	65.1	100.0
	**Total**	109	100.0	100.0	

*"Elderly people follow an exercise when encouraged by children or elderly friends. Even though they do not fully follow the exercise, they are glad because they receive food, meet their friends, talk and get treatment at the Public Health Center".*

Personal motivation, support from health workers and families, and acceptance of technology are behavior change techniques to increase the physical activity of elderly people [23].

### Quality of life

Table 3 shows that out of 109 elderly people, 69 (63.3%) elderly in Batik Nau District have a high level of life quality, while 40 elderly people (36.7%) have a low quality of life. Elderly people who do not exercise experience a decrease in physical fitness by 10–15% for each decade. Compared to those who regularly exercise, the decrease is only around 5–7% per decade [23]. The level of life quality of elderly people can be seen from a number of parameters, such as physical health, psychological health, social relationship, and environmental condition.

**Table 3.** The quality of life is based on physical health, psychology, social relationship, and environmental conditions.

		**Frequency**	**Percent**	**Valid percent**	**Cumulative percent**
**Valid**	Low	40	36.7	36.7	36.7
	**High**	69	63.3	63.3	100.0
	**Total**	109	100.0	100.0	

### Bivariate Analysis

#### The correlation between intrinsic motivation and the quality of life among the elderly

Bivariate analysis was used to identify the correlation between motivation (intrinsic and extrinsic) to participate in an exercise and the quality of life of the elderly. Data analysis was conducted using the Spearman Rank Correlation technique (categorical data). The result of the analysis is summarized in Table 4.

**Table 4.** The correlation between intrinsic motivation and the quality of elderly life during the Covid-19 pandemic.

**Symmetric Measures**				
	**Value**	**Asymptotic Standardized Error ^a^**	**Approximate T ^b^**	**Approximate Significance**
**Interval by Pearson's Interval R**	.400	.091	4.509	.000 ^c^
**Ordinal by Spearman Ordinal Correlation**	.400	.091	4.509	.000 ^c^
**N of Valid Cases**	109			

^a^ –Not assuming the null hypothesis; ^b^ – Using the asymptotic standard error assuming the null hypothesis; ^c^ – Based on normal approximation.

There was a significant correlation between intrinsic motivation and the quality of life among the elderly (r=0.40, p=0.000) (Table 4). This is in line with Yuliana, who stated that during the Covid-19 pandemic, elderly people needed assistance from all parties, family, health workers, and government. For example, exercise and sharing hobbies such as reading, watching a movie, and painting, because Covid-19 can decrease the quality of elderly life [25]. Humasfik mentioned that elderly people could do easy physical activity for 30 minutes and elderly exercise [26]. It is very useful for the elderly to improve endurance [27].

*"I am not strong enough to exercise because my arms and legs are no longer strong enough to move. I come to the exercise studio with children, just sitting around and talking with elderly people to reduce loneliness".* (BT, 68 years old).

In Nanggroe Aceh Darussalam, specifically in Titue Public Health Center, Pidie District, general factors, physical condition, and motivation impact as much as 30.7% of elderly exercise participation. 

*"I diligently participate in exercise because it makes me healthy, I can also sell food, and we are often invited to take a walk to sub-districts exercise competition. Even though the wages from selling food are small, I am glad to do it because I have things to do".* (FT, 61 years old).

Ada *et al.* research in Mojosongo village, Solo, Central Java, found that elderly people still working earn below the Regional Minimum Wage (UMR). The effort to meet the economic needs of the elderly people is not supported by their business ownership and skills, so they only rely on occupations that give a minimum wage [28]. 

The Ministry of Health vaccinated health workers, public officers, and elderly people against Covid-19. However, vaccination for the elderly, starting in February 2021, is running slower than others [29]. In 2019, Statistics Indonesia reported that as much as 9.4% of elderly people live by themselves. Due to deteriorating health, elderly people need a vaccination center that is affordable and close to their residence. Not all elderly people have a companion to take them to the vaccination center. This is one of the factors slowing down the elderly vaccination [30].

#### The correlation between extrinsic motivation and the quality of life among elderly

Table 5 shows a significant correlation (r=0.626, p=0.000) between extrinsic motivation and the quality of elderly life. Consequently, external factors in encouraging elderly people to participate in exercise will improve their quality of life. 

**Table 5.** The correlation between extrinsic motivation and the quality of elderly life.

**Correlation Coefficient Value (r-test) Symmetric Measures**				
	**Value**	**Asymptotic Standardized Error ^a^**	**Approximate T ^b^**	**Approximate Significance**
**Interval by Pearson's** **Interval R**	.626	.072	8.303	.000 ^c^
**Ordinal by Spearman Ordinal Correlation**	.626	.072	8.303	.000 ^c^
**N of Valid Cases**	109			

^a^ –Not assuming the null hypothesis; ^b^ – Using the asymptotic standard error assuming the null hypothesis; ^c^ – Based on normal approximation.

One study showed that the insomnia scale in elderly people begins to decline after doing exercise [31, 32]. The change in sleep quality among elderly people began to increase as much as 5.57% after exercising three times because it affects physical fitness. As conveyed by SR, a 70-year-old man:

*"I did not want to go to the exercise before. I did not believe that elderly exercise has a benefit. Because of my children's advice and motivation, I do it 3–4 times. Apparently, elderly exercise has benefits, in addition to sweating, joint pain is reduced, the blood pressure becomes normal, and I can sleep well".*

Lack of physical activity is one of the leading causes of obesity and functional disability in the elderly [33]. The primary purpose was to analyze the impact of motivational resistance training programs in meeting the health of elderly people. This is in line with the statement of AT (67 years old).

*"After I stopped participating in elderly exercise, my activities were only eating and sleeping. It makes my body fat, short of breath, and I often feel weak and fatigued".*

#### The correlation between motivation and the quality of life among the elderly during the Covid-19 pandemic

Table 6 shows a significant correlation (r=0,721, p=0,000) between motivation and the quality of life among the elderly, suggesting that the impact of internal and external factors in following exercise will improve the quality of life of elderly people. As conveyed by SY, a 75-year-old man: 

**Table 6.** The correlation between motivation and the quality of life among the elderly during the Covid-19 pandemic.

**Symmetric Measures**				
	**Value**	**Asymptotic** **Standardized Error ^a^**	**Approximate T ^b^**	**Approximate Significance**
**Interval by Pearson's** **Interval R**	.721	.069	10.769	.000 ^c^
**Ordinal by Spearman** **Ordinal Correlation**	.721	.069	10.769	.000 ^c^
**N of Valid Cases**	109			

^a^ –Not assuming the null hypothesis; ^b^ – Using the asymptotic standard error assuming the null hypothesis; ^c^ – Based on normal approximation.

*"At first, I did not want to leave the house: besides having difficulty walking, I also had a headache. After following the exercise accompanied by grandchildren, the headache is reduced, and my walk is no longer stiff. Because of the encouragement from my daughter-in-law and grandchildren, I can feel the benefit of participating in elderly exercise. I am so glad I can meet my old friends, but during the Covid-19, we do not do exercise anymore".*

## Discussion

A study conducted by Mora and Valencia in the United States suggested that regular physical activity is essential for healthy aging and can reduce chronic disease and prevent early death [34]. As many as 25% or 1.4 million people worldwide do not do physical activity, so many get some diseases. As stated by DW, a 65-year-old woman:

*"I am not actively participating in elderly exercise because there is no one to take me to a public health center. I cannot walk myself; my son-in-law uses the vehicle to work. If a neighbor is willing to pick me up, I follow the exercise. Because of the Coronavirus, the exercise is conducted at home. Even then, I do not do it, because I am not enthusiastic about doing an exercise myself. As a result, my body feels sick because it is lazy to move".*

Elderly people must be active in physical activity according to their abilities and condition. If not, it will increase the risk of chronic disease. Andesty and Syahrul mentioned that elderly people in UPTD Griya Werdha, Surabaya, have chronic diseases and bad social-relationship and quality of life [35]. There is a correlation between social interaction and the quality of elderly life, where the better social interaction the elderly have, the higher the quality of life. The head of the youth organization, AGU (46 years old) conveyed: 

*"In general, the elderly people in this village do not demand much. As long as they get a meal and drink and can-do worship, it is enough. If they miss their children and grandchildren, they can hear their voices through their cell phone. Simply, they live with their family, so it is reasonable if their quality of life is better because they get support from their family".*

This result does not differ much from a study conducted by Sitindaon and Yuliyana in Tanjung Pinang Anugrah Elderly Welfare Institution, Riau Islands [36]. There is a significant difference between the quality of elderly life after doing elderly exercise, which is that doing exercise improves the quality of elderly life.

## Conclusion

During the Covid-19 pandemic, the motivation of elderly people to participate in exercise was still high. Even though exercising in public health centers is prohibited, they still do physical activity at home. The quality of life of elderly people who live in the countryside is still high or in better condition because most of them do not have so many demands or pressure. Instead, they get adequate support from their family. Thus, only a few of them (in a small number) have a low quality of life. The only problem is the restriction that prevents them from practicing the exercise due to Covid-19. Therefore, they are hoping that public health officials will provide solutions so that the elderly can maintain their health without violating health protocols during the pandemic.

## Acknowledgments

### Conflicts of interest

The author declares no conflicts of interest.

### Ethical approval

Ethical clearance was obtained from the institutional ethical committee of Bengkulu University, Indonesia (4964/UN30.15/PP/2021),

### Consent to participate

Written informed consent was obtained from the participants in the study.

### Data availability

Further data is available from the corresponding author on reasonable request.

### Authorship

HM was responsible for collecting the data, writing, and analyzing the manuscript.
